# Unraveling How *Candida albicans* Forms Sexual Biofilms

**DOI:** 10.3390/jof6010014

**Published:** 2020-01-15

**Authors:** Austin M. Perry, Aaron D. Hernday, Clarissa J. Nobile

**Affiliations:** 1 Department of Molecular and Cell Biology, School of Natural Sciences, University of California, Merced, CA 95343, USA; aperry5@ucmerced.edu (A.M.P.); ahernday@ucmerced.edu (A.D.H.); 2 Quantitative and Systems Biology Graduate Program, University of California, Merced, CA 95343, USA

**Keywords:** biofilms, *Candida albicans*, sexual biofilms, pheromone-induced biofilms, mating type-like (*MTL*) locus, white cell, opaque cell, phenotypic states, pheromone signaling, biofilm formation, biofilm development

## Abstract

Biofilms, structured and densely packed communities of microbial cells attached to surfaces, are considered to be the natural growth state for a vast majority of microorganisms. The ability to form biofilms is an important virulence factor for most pathogens, including the opportunistic human fungal pathogen *Candida albicans*. *C. albicans* is one of the most prevalent fungal species of the human microbiota that asymptomatically colonizes healthy individuals. However, *C. albicans* can also cause severe and life-threatening infections when host conditions permit (e.g., through alterations in the host immune system, pH, and resident microbiota). Like many other pathogens, this ability to cause infections depends, in part, on the ability to form biofilms. Once formed, *C. albicans* biofilms are often resistant to antifungal agents and the host immune response, and can act as reservoirs to maintain persistent infections as well as to seed new infections in a host. The majority of *C. albicans* clinical isolates are heterozygous (**a**/α) at the mating type-like (*MTL*) locus, which defines *Candida* mating types, and are capable of forming robust biofilms when cultured in vitro. These “conventional” biofilms, formed by *MTL*-heterozygous (**a**/α) cells, have been the primary focus of *C. albicans* biofilm research to date. Recent work in the field, however, has uncovered novel mechanisms through which biofilms are generated by *C. albicans* cells that are homozygous or hemizygous (**a**/**a**, **a**/Δ, α/α, or α/Δ) at the *MTL* locus. In these studies, the addition of pheromones of the opposite mating type can induce the formation of specialized “sexual” biofilms, either through the addition of synthetic peptide pheromones to the culture, or in response to co-culturing of cells of the opposite mating types. Although sexual biofilms are generally less robust than conventional biofilms, they could serve as a protective niche to support genetic exchange between mating-competent cells, and thus may represent an adaptive mechanism to increase population diversity in dynamic environments. Although conventional and sexual biofilms appear functionally distinct, both types of biofilms are structurally similar, containing yeast, pseudohyphal, and hyphal cells surrounded by an extracellular matrix. Despite their structural similarities, conventional and sexual biofilms appear to be governed by distinct transcriptional networks and signaling pathways, suggesting that they may be adapted for, and responsive to, distinct environmental conditions. Here we review sexual biofilms and compare and contrast them to conventional biofilms of *C. albicans*.

## 1. Introduction

Biofilms are communities of microbial cells that are attached to surfaces and encased in a protective substance called the extracellular matrix [1,2,3,4,5]. Biofilms readily form on surfaces that are biotic (e.g., organs, mucosal and epithelial layers, and teeth) and abiotic (e.g., dentures, catheters, and industrial materials) [1,2,3,4,5]. The biofilm growth state provides the microorganisms inside with a sheltered microenvironment that is buffered against fluctuations in the surrounding environment and is protected from predators, environmental stresses, and mechanical forces that microorganisms would normally encounter in the planktonic (or free-living/free-floating) growth state [1,2,3,4,5]. Due to these adaptive benefits, most microorganisms under natural settings have evolved to spend the majority of their existence in the biofilm growth state [1].

Biofilm formation is a key virulence factor for most pathogens, including *Candida albicans,* which is the most commonly encountered human fungal pathogen in clinical settings [3,4,5,6,7]. *C. albicans* causes a wide variety of infections, ranging from benign mucosal (e.g., yeast infections and thrush) to hematogenously disseminated (bloodstream) candidiasis [6,7]. *Candida* infections are notably serious in immunocompromised individuals, such as AIDS patients, patients undergoing chemotherapy, transplantation patients receiving immunosuppression therapy, and patients with implanted medical devices [8,9,10]. Although research on *C. albicans* has been ongoing for over 70 years, most work has historically focused on *C. albicans* in its planktonic growth state. Over the last 20 years, however, the biofilm growth state of *C. albicans* has become a major area of research focus. *C. albicans* can form biofilms on abiotic surfaces (e.g., dentures, intravenous catheters, and prosthetic devices), as well as biotic surfaces (e.g., mucosal layers in the oral cavity and genitourinary tract) [3,4,5]. Once established, the cells within a *C. albicans* biofilm are protected from the host immune response, mechanical perturbations, and chemical stresses, allowing *C. albicans* to persist in the host and potentially cause recalcitrant infections [3,4,5]. More recently, a specialized “sexual” form of *C. albicans* biofilm has been discovered; although structurally similar to “conventional” biofilms, these “sexual” biofilms have many distinct phenotypic characteristics and generate a unique microenvironment that supports *C. albicans* mating [11,12].

Best known as the most common cause of life-threatening fungal infections in hospital settings, *C. albicans* is also a normal commensal in the majority of healthy humans. Remarkably, *C. albicans* can asymptomatically colonize several diverse regions of the body, including the oral cavity, gastrointestinal tract, skin, and genitourinary tract of humans [13,14,15,16]. These niches vary dramatically in terms of pH, nutrient sources and availability, and oxygen content [17,18]. This adaptive plasticity is due, in part, to the ability of *C. albicans* to undergo distinct morphological transitions in response to environmental cues; the best characterized examples include the yeast to hyphal cell transition and the transition between two distinct phenotypic cell types, termed “white” and “opaque” [5,18,19]. We begin by reviewing the white-opaque transition as it is intimately intertwined with the formation of sexual biofilms and mating. Next, we review the pheromone signaling and responses that occur in both white and opaque cell types during sexual biofilm formation and mating. Lastly, we compare and contrast conventional and sexual biofilms and consider the mechanisms through which sexual biofilms may aid in the process of mating.

## 2. The White–Opaque Transition

White and opaque cell types are heritably maintained for many generations, and reversible switching between the two cell states occurs stochastically under standard laboratory growth conditions [19]. This balance between the white and opaque states is influenced by specific environmental cues that can bias the switch towards one cell type or the other, or even force all of the cells in a population to adopt the white cell phenotype [18,19,20,21]. Approximately 16% of the genome is differentially expressed between the white and opaque cell types, resulting in cells with dramatically different phenotypes and functional attributes [18,22,23,24]. The morphologies of each cell type are also distinct; white cells are spherical and smooth and give rise to white, dome shaped colonies, whereas opaque cells are oblong and pimpled and form flatter and darker colonies [18,19]. Each state displays distinct metabolic preferences, resulting in striking fitness differences under a variety of environmental conditions [25]. White and opaque cells also respond to environmental conditions in unique ways; for example, opaque cells, but not white cells, can be induced to form filaments in response to nitrogen or phosphate limitation, while white, but not opaque cells, are induced to form filaments in the presence of serum [26]. The two cell types also display distinct responses to alterations in temperature under standard laboratory growth conditions; white cells are stable at 37 °C, while opaque cells revert to the white state en masse at 37 °C [18]. Opaque cells, however, can be stabilized at 37 °C by specific environmental stimuli, such as anaerobic conditions, elevated carbon dioxide levels, *N-*acetylglucosamine, or nutrient limitations [20,21,27,28,29,30,31]. Interestingly, each cell type also interacts with the host immune system in different ways; for example, white cells secrete a macrophage chemoattractant while opaque cells do not, thus increasing the likelihood for opaque cells to escape macrophage engulfment, possibly allowing them to evade this aspect of the host innate immune response [32].

The ability to undergo the white to opaque transition is controlled by the configuration of a discrete region on chromosome 5 known as the Mating Type-Like (*MTL*) locus [33,34,35]. The *C. albicans MTL* locus can carry two distinct configurations, **a** and α, which consist of genes that specify the **a** and α mating types, respectively [35]. Most *C. albicans* clinical isolates (~97%) are diploid and exist in the *MTL*-heterozygous (**a**/α) configuration, however a few clinical isolates have been found to exist in the *MTL*-homozygous (**a/a** or α/α) configuration [33,34]. *MTL*-heterozygous strains express the sex genes *MTL**a**1* and *MTLα2,* the protein products of which form a heterodimer that directly represses the white to opaque transition by binding to the promoter region of *WOR1,* the master regulator of the opaque cell type, and repressing its transcription [33,36]. *MTL*-homozygous strains contain either *MTL**a**1* or *MTLα2*, but not both, and thus *WOR1* expression is derepressed and switching to the opaque state occurs stochastically at a rate of approximately once every 10^4^ cell divisions [19,33,34,36]. The white state is considered to be the default cell type, and is often referred to as the “ground state” of the white-opaque switch, since it does not require activation of any known white to opaque transition regulators, while the opaque state is referred to as the “excited state” of the switch because it requires expression of Wor1, which results in activation of many additional regulatory and non-regulatory genes that are specific to the opaque state [22,37].

Although the vast majority (~97%) of *C. albicans* clinical isolates are heterozygous at the *MTL* locus, and were previously presumed to be “locked” in the white cell state [33,34], recent research has shown that the white to opaque switch may be a much more common occurrence in vivo than previously thought. For example, it is now appreciated that natural *MTL*-heterozygous isolates can undergo white to opaque switching in vitro under elevated levels of CO_2_ and in the presence of *N*-acetylglucosamine, conditions that resemble that of the gastrointestinal tract; however, unlike *MTL*-homozygous opaque cells, these *MTL*-heterozygous opaque cells appear unable to mate [21]. In *MTL*-heterozygous cells, *HBR1*, which encodes a transcription factor that mediates the hemoglobin response, promotes expression of genes carried at the *MTLα* locus and thus indirectly reinforces the **a**1/α2-mediated repression of *WOR1* and ultimately the repression of white to opaque switching [38,39]. Deletion of one copy of *HBR1* in *MTL*-heterozygous cells results in a substantial reduction in *MTLα1* and *MTLα2* mRNA expression levels and a slight upregulation of *MTL**a**1* gene expression; the resulting reduction in **a**1/α2 heterodimer levels allows these cells to behave like **a** cells in regards to switching and mating [38,39]. In another example, deletion of *OFR1*, which encodes a protein of unknown function, enables *MTL*-heterozygous white cells to switch to the opaque state and express both **a**- and α-specific pheromones and pheromone receptors, conferring *ofr1* mutants with the unique ability to mate with opaque cells of any *MTL* configuration [40]. In addition, an *MTL*-homozygous clinical isolate strain P94015, which was observed to drift between “white-like” and “opaque-like” cell states, was found to contain a homozygous nonsense mutation in *EFG1*, which encodes a known repressor of the white to opaque transition [41]. Taken together, physiologically relevant environmental cues, or spontaneously arising loss-of-function mutations, could enable naturally occurring strains to undergo white to opaque switching. Lastly, *MTL*-heterozygous cells can become *MTL*-homozygous through loss of heterozygosity on part or all of chromosome 5. This can occur through local gene conversion, homozygosis of an entire arm of the chromosome, or through spontaneous loss of one copy of chromosome 5 followed by duplication of the remaining homologous chromosome [42,43]. These loss of heterozygosity events have been shown to occur in response to a wide range of environmental conditions, including exposure to antifungal agents, growth in the presence of sorbose, oxidative stress, and temperature stress [42,43,44,45,46].

In addition to *MTL*-heterozygous cells becoming *MTL*-homozygous, *MTL*-homozygous cells can also become *MTL*-heterozygous through the *C. albicans* parasexual life cycle [47]. During parasex, *MTL*-homozygous opaque cells can become *MTL*-heterozygous by mating with *MTL*-homozygous cells of the opposite mating type; this is termed heterothallic mating [47,48,49]. Interestingly, opaque cells can also mate with opaque cells of the same mating type, termed homothallic mating, providing a means for genetic exchange within unisexual populations and even between clonal progeny of a single parent cell [50]. Generally, the parasexual life cycle requires that *MTL*-heterozygous white cells undergo loss of heterozygosity at the *MTL* locus followed by switching to the opaque cell state [33,51,52,53]. The resulting *MTL*-homozygous opaque cells secrete sex-specific pheromones that can cause opaque cells of the opposite mating type to extend mating projections towards the highest pheromone concentration gradient [53]. Once two mating projections fuse, the resulting conjugation bridge allows for nuclear fusion and the formation of a tetraploid nucleus [53]. This structure is stable for several cell divisions, thereby producing tetraploid progeny [49,53]. Specific environmental cues can cause the tetraploid cells to reduce their ploidy state via concerted chromosome loss, thereby completing the parasexual life cycle by producing diploid cells [48,49,54,55]. This concerted chromosome loss, however, can often result in aneuploidy, which is hypothesized to allow *C. albicans* to rapidly adapt to variable environments and harsh conditions [49,55]. While asexual reproduction (e.g., through budding) can benefit *C. albicans* populations by preserving well-adapted genotypes, parasex can generate novel allelic combinations to allow for rapid evolution in changing environments [48,54,55], which may contribute to the remarkable ability of *C. albicans* to colonize diverse niches in the body and to its overall success as a commensal and pathogen [49]. Despite these apparent benefits, parasex has thus far been reported to occur at low rates in vivo [27,47]. Given that ~97% of the *C. albicans* population in vivo is thought to be *MTL*-heterozygous [34], the probability that two *MTL*-homozygous white cells of opposite mating types undergo the multiple steps required for mating simultaneously, and within close enough proximity to detect mating pheromone, seems exceedingly low. Recent research, however, is beginning to uncover that homothallic mating occurs more frequent under specific in vitro environmental conditions, such as glucose starvation and oxidative stress, supporting the idea that homothallic mating may be more common than anticipated in vivo [56]. Intriguingly, parasexual mating is hypothesized to occur at high frequencies within sexual biofilms, which are formed by *MTL*-homozygous white cells in response to mating pheromone [11,12]. Like all *C. albicans* biofilms, the multilayer structure of the sexual biofilm is such that its innermost layers are likely to contain lower levels of oxygen and nutrients than the layers closer to its surface, and thus sexual biofilms could be a niche that supports homothallic mating.

Perhaps the most striking difference between the white and opaque cell types is that opaque cells can mate with other opaque cells, but form severely impaired biofilms, while white cells can form robust biofilms, but are unable to mate [11,12,33,47,52,57,58]. Generally, a *C. albicans* biofilm consists of a basal layer of yeast cells with hyphae and pseudohyphae extending away from the substrate to which they are adhered [5,59,60]. In recent years, it has been shown that *MTL*-heterozygous and *MTL*-homozygous white cells form different types of biofilms in response to different stimuli [11,12,57,58,59]. *MTL*-heterozygous (**a**/α) cells form robust biofilms in response to shear flow forces and various environmental conditions (e.g., temperature, shifts in pH, etc.), and are termed conventional biofilms [5]. Once formed, conventional biofilms are challenging to treat in clinical settings due to their recalcitrance to antifungal agents, mechanical forces, and the host immune response. Alternatively, sexual biofilms formed by *MTL*-homozygous (**a** or α) white cells in response to mating pheromone are less robust than conventional biofilms [11,12], but as discussed above, they may provide an adaptive niche for mating.

## 3. Pheromone Signaling and Response

### 3.1. Mating Pheromones

The **a** and α pheromones produced by *C. albicans*, encoded by *MF**a**1* and *MFα1* respectively, play essential roles in the processes of heterothallic and homothallic mating [50,61,62,63]. Opaque α cells constitutively express high levels of *MFα1*, producing a trimeric pheromone precursor peptide, whereas white α cells do not [62]. This α-pheromone precursor peptide is thought to be post-translationally modified by the Kex2 protease and Ste13 dipeptidyl aminopeptidase A, to result in two secreted and identical tridecapeptides with the sequence GFRLTNFGYFEPG and one tetradecapeptide with the sequence GFRLTNFGYFEPGK that represent the mature α pheromones; both the tridecapeptide and tetradecapeptide are capable of eliciting mating responses [62,63,64,65,66,67,68]. In contrast, **a** cells only weakly express *MF**a**1* under standard laboratory conditions [61]. However, when exposed to α-pheromone, white and opaque **a** cells highly express both *MF**a**1* and *MFα1* [50,58]. *MF**a**1* also encodes a precursor peptide which is predicted to be processed similarly to the **a**-pheromone of *Saccharomyces cerevisiae* [61,69,70]. Initial cleavage from the **a** pheromone precursor peptide is thought to occur via the Ste24 and Axl1 proteases [61,69]. The developing peptide is then further processed by the prenyl-group-adding enzymes Ram1 and Ram2, the prenyl-dependent protease Rce1, and the cysteine-carboxy methyltransferase Ste14 [61,69]. The mature **a**-pheromone is a prenylated tetradecapeptide with the sequence AVRSVSTGNCCSTC, and requires Hst6, an ABC transporter, to leave the cell [61,70,71]. Due to the structural simplicity of α-pheromone and the fact that α-pheromone can be more easily chemically synthesized relative to **a**-pheromone, most pheromone signaling experiments in the field are carried out using **a** cells and the addition of chemically synthesized α-pheromone.

Although both *MF**a**1* and *MFα1* are expressed in **a** cells in response to pheromone, α-pheromone is typically degraded by Bar1, an aspartyl protease, via a phenomenon known as “barrier activity” [72]. Barrier activity promotes heterothallic mating in ascomycetes by preventing pheromone hyperstimulation and by allowing for a recovery from cell cycle arrest [72]. It also inhibits the ability of *C. albicans* to undergo auto-pheromone stimulation and thus prevents homothallic mating. Deletion of *BAR1* in *C. albicans* allows for homothallic mating through an autocrine pathway where opaque **a** cells excrete α-pheromone, which then binds to Ste2, the α-pheromone receptor, on the same cell, leading to self-activation for mating [50]. In addition, glucose starvation and oxidative stress enable unisexual populations of opaque **a** cells to undergo homothallic mating despite high *BAR1* expression levels [56], resulting in auto-activated opaque cells that can mate with other opaque cells of the same mating type [50,56]. These findings suggest that certain strain backgrounds as well as specific niches in the human body can override the phenomenon of barrier activity, allowing for unisexual populations to become activated by pheromone [50,56]. This has important consequences for the parasexual lifecycle of *C. albicans* as homothallism allows for same-sex mating to occur within cell mixtures of the same mating types and between certain strains that are incompatible for heterothallic mating [50]. Given that this mechanism results in pheromone stimulation and mating for unisexual populations of opaque cells, a similar scenario could be envisioned within a sexual biofilm. The biofilm environment may even enhance the rate of homothallic mating by sequestering pheromone and possibly protecting pheromone from degradation within the biofilm structure [11,12]. In addition, within a biofilm, recently divided opaque cells would be held in close proximity to each other, increasing both the likelihood of finding a mate nearby and the frequency of mating between progeny of a single opaque cell. Given that *C. albicans* relies on generating aneuploid progeny for genetic diversity, rather than recombination during meiosis, homothallic mating between clones in this capacity could rapidly and efficiently introduce genetic diversity into a population [50,54,55].

### 3.2. Pheromone-Signaling Pathway Control

*C. albicans* employs a conserved Mitogen-Activated Protein Kinase (MAPK) signaling pathway to transduce pheromone signals and alter gene expression [73,74]. This pathway begins with the conserved mating type-specific G-protein coupled receptors (GPCRs), Ste2, expressed on **a** cells to recognize α-pheromone, and Ste3, expressed on α cells to recognize **a**-pheromone [73,74,75]. Activation of either GPCR results in the dissociation of the G_α_ subunit (Cag1) from the G_β_ subunit (Ste4), and the G_γ_ subunit (Ste18) of a heterotrimeric G-protein [73,74,75,76]. The G-protein subunits then activate Cst20, a kinase that activates the downstream MAPK cascade, consisting of Ste11, Hst7, and Cek1/Cek2 [73,74,75]. All kinases in this pathway, with the exception of Cst20, are held together in close proximity by the scaffolding protein Cst5 [73,74,75,77]. Cek1 and Cek2 then activate the transcription factor Cph1 in both white and opaque cells, resulting in the differential expression of white and opaque state-specific genes [58,73]. The activities of Cek1 and Cek2 are regulated by Cpp1, a MAP kinase phosphatase [78]. Interestingly, *STE4*, *CST5*, *CEK1,* and *CEK2* are expressed at lower levels in white cells than opaque cells [79], and their repression contributes to the sterility of white cells as white cells engineered to express *STE4*, *CST5,* and *CEK2* (*CEK1* was not tested) at levels similar to opaque cells have been shown to undergo mating at frequencies approaching that of opaque cells [79]. It is also interesting to note that Cek1 (rather than Cek2) appears to play a major role in opaque cell mating; opaque *cek1* mutants mate at much lower frequencies than opaque *cek2* mutants [78]. The precise contributions of Cek1 and Cek2 to the pheromone response in white and opaque cells is complex and an intriguing research area for future study. Nonetheless, we do know that G-protein signaling pathways, such as this one, are highly conserved among fungal pathogens and are involved in controlling several important developmental processes, including mating, filamentation, and virulence [80].

### 3.3. Differences Between the White and Opaque Cell Pheromone Responses

When opaque cells sense pheromone of the opposite mating type, they become activated for mating via the MAPK signaling pathway (described above). This pheromone stimulation can occur under a variety of different environmental conditions, including planktonic and biofilm conditions [58,61,62,68]. Interestingly, opaque cells have been observed to respond more efficiently to pheromone in media containing alternative carbon sources (e.g., Spider media) [68]. Additionally, the opaque cell pheromone response can be enhanced under a variety of environmental conditions by deletion of *GPA2,* which encodes a G-protein α-subunit that functions at the beginning of the cyclic AMP-protein kinase A (cAMP-PKA) pathway [68]. This finding suggests that mating may occur more frequently within certain (e.g., specific nutrient limiting) host niches and that there is likely crosstalk between the signaling pathways regulating pheromone (i.e., MAPK) and nutrient sensing (i.e., cAMP-PKA) responses.

**Figure 1:**
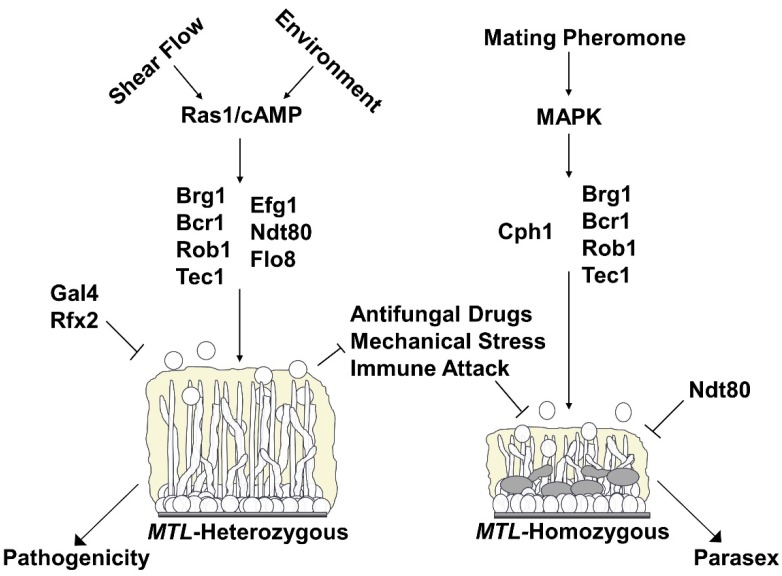
Summary of the regulation of *C. albicans* conventional (mating type-like (*MTL*)-heterozygous) and sexual (*MTL*-homozygous) biofilm formation and their phenotypic characteristics. Arrows with smaller heads indicate activation (e.g., shear flow and environmental conditions activate the Ras1/cAMP pathway). Arrows with large heads indicate the lifestyle each biofilm type facilitates (e.g., *MTL*-heterozygous biofilms facilitate a pathogenic lifestyle). T-bars indicate inhibitory relationships (e.g., Gal4 and Rfx2 inhibit conventional biofilm formation and conventional biofilms inhibit the deleterious effects of antifungal drugs, mechanical stress and immune attack).

The opaque cell pheromone response in *C. albicans* is mediated by the transcription factor Cph1, a homolog of the transcription factor Ste12 in *S. cerevisiae* that is activated by a MAPK signaling pathway and controls genes involved in mating [58,70,73,74,81,82,83]. In opaque cells responding to pheromone, Cph1 initiates a transcriptional response that results in an upregulation of genes involved in filamentation (e.g., *FGR23*), cell fusion (e.g., *FUS1*, *FIG1*), karyogamy (e.g., *KAR4*), MAPK signaling (e.g., *CEK1/2*), and adhesion and virulence (e.g., *HWP1/2*, *ECE1*, *SAP4/5/6*, *RBT1/4*) [52,53,58,62,68]. Interestingly, although opaque cells generally grow slower than white cells, genes involved in DNA replication and the cell cycle (e.g., *MCM6*, *MCM7*, *PRI2*, and *POL5a*) are specifically repressed in opaque cells responding to pheromone, suggesting that exposure to pheromone further slows progression out of the G1 phase of the cell cycle [52,53,58,62,68,84]. In opaque **a** cells, *STE2* is upregulated, and the α-pheromone receptor Ste2 becomes localized to the tip of growing cellular extensions known as mating projections or conjugation tubes [11,52,53,58,62]; mating projections are phenotypically similar to hyphae, but lack septa [52,53]. Not surprisingly, transcriptional profiling data revealed that opaque cells forming mating projections in response to pheromone upregulate a subset of the genes associated with filamentation and virulence that are upregulated by white cells forming hyphae in response to serum [62,85]. These findings indicate that there is overlap among genes expressed during hyphal formation and pheromone treatment, but that there are also several genes that are distinctly expressed in each process [62].

Although *C. albicans* white cells are unable to mate, **a** and α white cells still express pheromone receptors and are thought to respond to pheromone in a Cph1-dependent manner [11,58], albeit at a much slower rate than opaque cells [58]. For example, under standard sexual biofilm conditions, the transcriptional response of opaque cells four hours after pheromone exposure is comparable to that of white cells 24 h after pheromone exposure [58]. Interestingly, the pheromone response in white cells appears to occur primarily under sexual biofilm conditions; in fact, much of the response is lost when white cells are subjected to pheromone under planktonic conditions [52,68]. It is also interesting to note that similar to the pheromone response in opaque cells, sexual biofilm formation is highly dependent on nutrient levels [57,68,86], suggestive again of crosstalk between the pheromone response and nutrient sensing signaling pathways. Despite white cells being unable to mate, many genes involved in MAPK signaling and mating are upregulated in white cells responding to pheromone (e.g., *STE2*, *HST6*, *FIG1*, *FUS1*, *KAR4*), which may be an artefact derived from the co-option of Cph1 by white cells for biofilm formation [58]. In addition, many of the adhesion-, biofilm- and other virulence-associated genes upregulated in opaque cells responding to pheromone are similarly upregulated by white cells responding to pheromone in biofilms (e.g., *RBT1*, *HWP1/2*, *ECE1*, *PGA23/50*, *SAP5/6*) [58]. However, unlike opaque cells, white **a** cells do not experience a halt in their cell cycle upon exposure to α-pheromone [11,52]. Overall, in synthetic pheromone-stimulated biofilms, 116 genes are differentially expressed in both white and opaque cells, white cells uniquely differentially express 147 genes, and opaque cells uniquely differentially express 190 genes [58]. Given that Cph1 is believed to mediate both sexual biofilm formation in white cells and mating in opaque cells in response to pheromone, Cph1 may be involved in mediating a core pheromone response involving filamentation and adhesion that can be modified depending on the epigenetic state of the cell [58,73]. Over the course of evolutionary time, it appears that *C. albicans* has rewired aspects of cell–cell communication to be used for host–pathogen interactions, which may provide insight into the unique history of this opportunistic pathogen. Additional work on the regulatory controls of white and opaque cells may improve our understanding of how transcription factors drift to regulate novel functions.

## 4. Conventional and Sexual Biofilms

### 4.1. Properties of Conventional and Sexual Biofilms Compared

Conventional and sexual biofilms formed by *C. albicans* are both composed of yeast-form, pseudohyphal, and hyphal cells [5,12,60,86]. The *C. albicans* biofilm life cycle typically begins when planktonic yeast-form cells adhere to a substrate in response to specific environmental stimuli [4,5]. These yeast-form cells proliferate, resulting in a dense mat that is tightly anchored to its substrate. Hyphae and pseudohyphae then begin to grow and extend away from the substrate, providing architectural support for the biofilm. As the growing *C. albicans* biofilm matures, the cells within the biofilm produce extracellular matrix material, composed predominantly of proteins, polysaccharides, and DNA that surrounds all of the cells within the biofilm [4,5]. Once a mature biofilm is formed, daughter yeast-form cells disperse from the biofilm and revert to the planktonic growth state or form new biofilms elsewhere [4,5,87]. Although this generalized biofilm life cycle is common across all *C. albicans* biofilms, the configuration of the *MTL* locus and the phenotypic state of the cells play important roles in determining the environmental stimuli that induce biofilm formation as well as certain unique physical characteristics of the biofilms formed. *MTL*-heterozygous white cells form thick and resilient conventional biofilms in response to specific environmental stimuli, such as shear flow rate and host factors, whereas *MTL*-homozygous white cells form thinner and weaker sexual biofilms in response to mating pheromone [11,12,57,86].

Generally, microorganisms that exist in biofilms are protected from environmental stresses relative to microorganisms that exist planktonically [1]. The extracellular matrix surrounding both *C. albicans* conventional and sexual biofilms acts as a physical barrier inhibiting many compounds, such as antimicrobial agents, from penetrating into the deeper layers of the biofilm [3,4,5]. Mature conventional biofilms, in particular, are highly resilient to most forms of environmental stress, such as treatment with antifungal agents, exposure to mechanical forces, and attack by the host immune system [4,5]. In addition to the physical barrier provided by the matrix, the resilience of conventional biofilms to antifungal agents is also due to the fact that cells within conventional, but not sexual, biofilms upregulate drug efflux pumps (e.g., Cdr1/2, Mdr1), thereby prohibiting antifungal drugs from reaching lethal concentrations within the biofilm [58,60]. Consistent with this finding, sexual biofilms are much more easily permeated by a variety of compounds than conventional biofilms [12,59]. Interestingly, this phenotype can be partially rescued by the overexpression of *BCR1* [12,59], which encodes the biofilm master regulator of several downstream adhesins, suggesting that cell–cell and/or cell–substrate adherence may also contribute to the recalcitrance of conventional biofilms to antimicrobial compounds. Cells within conventional biofilms are also more tightly adhered to each other and their substrates compared to sexual biofilms [12,58,59]. These differences in adherence are likely due to the upregulation of genes involved in adhesion (e.g., *ALS3*) in conventional biofilms, which are less (if at all) upregulated in sexual biofilms [3,4,5,58,60]. Additional factors contributing to the drug resistance of conventional biofilms include variation in cell membrane sterol composition and the presence of metabolically dormant persister cells, which can display extreme tolerance to most classes of antifungal drugs [3,4,13,88,89]. We note that these two factors have only been studied in conventional biofilms, and thus whether or not they also are present in sexual biofilms is unknown, and an intriguing area of interest for future studies.

If sexual biofilms do not provide the same protective environment as conventional biofilms, why does *C. albicans* bother to form sexual biofilms in the first place? Given that ~97% of the *C. albicans* population in nature is thought to be *MTL*-heterozygous, the chance that two *MTL*-homozygous white cells of opposite mating types will exist in close enough proximity to undergo the complex steps involved to mate is seemingly unlikely [34]. Even if two opaque cells were in close enough proximity to one another, ambient forces would likely disrupt the pheromone concentration gradient before the cells could find one another and fuse. Since sexual biofilms are not nearly as thick or dense as conventional biofilms, these properties could enable opaque cells to extend mating projections through the biofilm towards other opaque cells, while still being sufficiently dense to maintain pheromone gradients and provide some stability against external forces [11,12]. Consistent with the idea that sexual biofilms provide an optimal environment for mating, white **a** cells produce their own pheromone when responding to α-pheromone, which promotes both homothallic and heterothallic mating [90]. In terms of the host response, white cells are preferentially phagocytosed by macrophages as compared to opaque cells and only white cells secrete a leukocyte chemoattractant [32,91]. Thus, white cells may protect mating opaque cells by acting as decoys to sequester infiltrating host cells [32]. Overall, by stabilizing pheromone gradients and providing an optimal environment for opaque cells to undergo mating, sexual biofilms may promote mating in specialized niches of the body that support white-opaque switching (e.g., the skin).

Cell heterogeneity resulting from the various microenvironments present throughout conventional biofilms is also likely to contribute to biofilm resilience [3]. These microenvironment differences lead to specific gene expression changes within cells in discreet environmental niches of the biofilm, resulting in widespread cellular heterogeneity throughout the biofilm architecture [92]. For example, the innermost regions of conventional biofilms are hypoxic and contain less nutrients and more waste products compared to the outermost regions of the biofilm [93]. These unique microenvironments also enable *C. albicans* to coexist and interact with specific microbial species. For example, the hypoxic inner regions of conventional *C. albicans* biofilms support the growth of obligate anaerobic bacteria, such as *Bacteroides fragilis* and *Clostridium perfringens* [3,93]. Although the microenvironments present in sexual biofilms have not been studied to date, because sexual biofilms are much thinner than conventional biofilms [12,59], there are likely to be fewer opportunities for microenvironments to form. Nonetheless, given their phenotypic differences, the microenvironments of conventional and sexual biofilms are certainly distinct.

Interspecies interactions within polymicrobial biofilms between *C. albicans* and other species (mostly bacteria) have only been studied to date within the context of conventional *C. albicans* biofilms. These interactions can be beneficial or antagonistic in nature. A large proportion of research to date has focused on the beneficial interactions between *C. albicans* and *Staphylococcus* species, such as *Staphylococcus aureus*; these two species are often co-isolated from biofilm infections with high mortality rates in clinical settings [94]. Although these two species can form biofilms independently, initial attachment of *C. albicans* cells to surfaces is enhanced when *C. albicans* is co-inoculated with *S. aureus* [95]. In mature polymicrobial biofilms of *S. aureus* and *C. albicans*, *S. aureus* cells can be found adhered to *C. albicans* hyphae and are present throughout the biofilm structure [95,96,97]. *S. aureus* is, in fact, known to specifically recognize and bind to the adhesin Als3 on the cell surface of *C. albicans* hyphae, and consistent with this, cells of *C. albicans als3* mutants have been found to interact with significantly fewer *S. aureus* cells than wild-type *C. albicans* cells [96]. Interestingly, *ALS3* expression is reduced in sexual biofilms compared to conventional biofilms [58,60], and thus one may hypothesize that *S. aureus* and *C. albicans* are less likely to co-localize in the context of sexual biofilms. Other structural components of *C. albicans* biofilms are also known to play roles in mixed-species interactions. For example, β-glucans present in the extracellular matrix of *C. albicans* biofilms were found to aid methicillin-resistant *S. aureus* (MRSA) strains in surviving vancomycin, one of the few antibiotics effective against MRSA [3,98]. In terms of antagonistic interactions, *Enterococcus faecalis* can secrete EntV, a bacteriocin that inhibits conventional *C. albicans* biofilm formation [3,99]. In another example, *Pseudomonas aeruginosa* can secrete a 12-carbon acyl homoserine lactone that hinders *C. albicans* filamentation and conventional biofilm formation by mimicking farnesol, a quorum sensing molecule produced by *C. albicans* that modulates filamentation [100,101]. *P. aeruginosa* can also release phenazines that specifically inhibit *C. albicans* filamentation and conventional biofilm formation [102]. Overall, given that sexual and conventional biofilms have different physical and biochemical properties, the interactions of these two biofilm systems with other microorganisms are likely to differ considerably.

Conventional and sexual biofilms also differ in their interactions with the host immune response. Neutrophils and mononuclear leukocytes are important host players against fungal infections [103,104]. When neutrophils recognize *C. albicans* cells, they activate a number of antimicrobial defenses, including phagocytosis, degranulation, the release of reactive oxygen species (ROS), and the release of web-like neutrophil extracellular traps (NETs) [103]. In general, neutrophils are very effective at killing planktonic *C. albicans* yeast and hyphal cells [105], where these antimicrobial mechanisms work efficiently. When it comes to *C. albicans* conventional biofilms, however, neutrophils are generally unable to penetrate beyond the outermost regions of the biofilm, ROS are not produced, and NETs are not released [3,4,59,106,107]. This biofilm-specific recalcitrance to neutrophils is largely due to the presence of the extracellular matrix, as physical disruption of the matrix in conventional biofilms restores the ability of neutrophils to release NETs [106]. Consistently, neutrophils are able to release NETs and kill *C. albicans* cells within a biofilm formed by the *C. albicans pmr1* mutant, which is unable to produce matrix mannan [106]. Interestingly, in the presence of a sexual biofilm, neutrophils can penetrate into the innermost layers of the biofilm [59], although whether NETs are released, and fungal cells are killed is unknown. Based on this information, one would hypothesize that sexual biofilms are more susceptible to clearance by neutrophils than conventional biofilms.

In terms of mononuclear leukocytes, these host cells typically respond to *C. albicans* infection by phagocytosing invading cells and releasing cytokines [108]. *C. albicans* cells in conventional biofilms are two to three times more resistant to killing by mononuclear leukocytes than cells growing planktonically [103,108]. In addition, *C. albicans* cells growing in conventional biofilms are capable of altering the cytokine profile of attacking mononuclear cells [108]. For example, the presence of a conventional biofilm leads to the downregulation of TNF-α, a pro-inflammatory cytokine produced by mononuclear leukocytes that would normally suppress biofilm formation [103,108,109]. Intriguingly, conventional biofilms that are grown in the presence of mononuclear leukocytes form thicker biofilms, a phenomenon that is thought to be mediated by an unknown soluble factor that is present when the two are co-cultured [108]. Whether or not this process also occurs with sexual biofilms in the presence of mononuclear cells is unknown, but an interesting area for future exploration.

The host response to *C. albicans* infection is typically initiated by the interaction of host pattern recognition receptors and pathogen-associated molecular patterns (PAMPs) and involves secretion of a variety of antimicrobial compounds. Interestingly, several characteristics of conventional and sexual biofilms inhibit the recognition of PAMPs. For example, hyphal cells, a major component of both conventional and sexual biofilms, are able to ‘mask’ the β-glucan in their cell walls, blocking a key PAMP recognized by many host immune cell types [4,110,111]. In addition, several cell surface and secreted proteins are capable of sequestering and inactivating host complement proteins, and other secreted anti-immune proteins are expressed at higher levels in conventional biofilms than in planktonic cells [3,4,60]. Although studies to date have only examined conventional biofilms, it seems likely that sexual biofilms would also retain some of these host response characteristics. In fact, we know that some cell surface and secreted proteins involved in inactivating the host immune response (e.g., *SAP4, MSB2*) are also upregulated in sexual biofilms [58]. Nonetheless, how sexual biofilms interact with the immune system and how they compare to conventional biofilms in this regard has not been investigated to date.

### 4.2. Genetic Regulation of Conventional and Sexual Biofilms

Our current knowledge of the regulation of conventional and sexual biofilms is summarized in Figure 1. Given that there are many phenotypic differences between conventional and sexual biofilms, it seems likely that the genetic regulation and transcriptional profiles of these two systems should differ as well. As discussed above, the signaling pathway that triggers the formation of sexual biofilms is a MAPK cascade initiated by the pheromone receptors Ste2 or Ste3 [73,74,75]. This pathway is unique to sexual biofilms, as a Ras1/cAMP pathway that includes Cdc35, Tpk2, and an unknown receptor has been shown to trigger conventional biofilm formation [59,112,113]. In the conventional biofilm pathway, Ras1 activation results in cAMP production, and increased concentrations of cAMP stimulate PKA to initiate the complex transcriptional network controlling conventional biofilm formation [59,112,113]. When comparing the transcriptional profiles of *MTL-*heterozygous white cells grown planktonically versus in conventional biofilm conditions, and white **a** cells grown in sexual biofilm conditions with and without the presence of α-pheromone, there are 662 genes that are induced twofold or more in conventional biofilms, 486 genes that are induced twofold or more in sexual biofilms, and 128 genes similarly induced twofold or more in both systems (examples include *HWP1*, *SAP4*, *SAP5*, *ALS1*) [58,60]. In addition, 187 genes are repressed at least twofold in conventional biofilms, 355 genes are repressed at least twofold in sexual biofilms, and only 19 genes are similarly repressed at least twofold in both systems [58,60]. The dramatic differences in transcriptomic profiles between sexual and conventional biofilms strongly supports the idea that distinct transcriptional networks regulate the formation of these two structures.

The core transcriptional network controlling conventional biofilm formation consists of nine transcription factors: Tec1, Ndt80, Rob1, Brg1, Bcr1, Efg1, Flo8, Gal4, and Rfx2 [60,114]. By screening a mutant library containing 165 strains with homozygous deletions of genes encoding DNA-binding proteins, a transcriptional network of six transcription factors was identified (Tec1, Ndt80, Rob1, Brg1, Bcr1, Efg1), whose deletion hindered conventional biofilm formation in vitro and in vivo [60]. Interestingly, two of these transcription factor mutants were defective in one in vivo model of biofilm formation but not in another (e.g., the *bcr1* mutant was severely defective in the rat catheter model, but formed a decent biofilm in the rat denture model, while the *brg1* mutant formed normal biofilms in the catheter model, but was severely defective in the denture model) [60]. These findings suggest that the genetic regulation of conventional biofilms may be different depending on the environment [60]. Further investigation into the transcriptional regulators of conventional biofilm formation in a temporal biofilm study revealed three additional core regulators: Flo8, Gal4 and Rfx2 [114]. Interestingly, deletion of *GAL4* and *RFX2* resulted in generally enhanced conventional biofilms relative to wildtype, indicating that they may serve as negative regulators of the network [114]. In order to understand how these transcription factors regulate conventional biofilm formation, genome-wide chromatin immunoprecipitation and microarray experiments were performed on each transcription factor and transcription factor mutant, respectively. These experiments revealed that each of the nine transcription factors contribute to the formation of a complex network that encompasses about 1000 downstream “target” genes [60]. Furthermore, extensive binding between the nine transcription factors and their respective *cis*-regulatory regions highlights a complex set of regulatory feedback loops within the core of the biofilm regulatory network [60,114,115]. Overall, the majority of TFs involved in the conventional biofilm network act as both positive and negative regulators of various downstream target genes, with the exception of Tec1, which seems to act primarily as an activator [60]. Although the core transcriptional network regulating conventional biofilm formation has been identified, many additional transcription factors have been found to regulate certain aspects of conventional biofilm formation. For example, Rlm1 and Zap1 are both involved in the regulation of the extracellular matrix [116,117,118]. As we continue research on biofilms into the future, there will certainly be additional regulators identified to play important roles in different aspects of conventional biofilm formation, as well as an increase in our knowledge of the core regulators of sexual biofilm formation.

Sexual biofilms are currently known to rely on four of the nine core transcription factors involved in the conventional biofilm network: Bcr1, Rob1, Brg1, and Tec1 [58]. Deletion of any of these four transcription factors results in a significant decrease in sexual biofilm thickness relative to wildtype [58]. Deletion of *EFG1* does not hinder sexual biofilm formation [58]; rather, the *efg1* mutant appears to form equally thick sexual biofilms relative to wildtype, indicating that *EFG1* is not required for sexual biofilm formation [58]. Interestingly, the *ndt80* mutant forms thicker sexual biofilms than wildtype, although this may not be due to Ndt80 acting as a negative regulator of sexual biofilm formation since deletion of *NDT80* leads to the misregulation of cell separation genes, specifically *SUN41* and *CHT3* [58]. This could consequently result in thicker sexual biofilms as an artifact of enhanced cell clumping and/or reduced cell dispersion during sexual biofilm formation. The fact that this does not occur in conventional biofilms, and that Ndt80 is in fact required for conventional biofilm formation, is an intriguing area for future research. The roles of the other three core transcription factors involved in regulating conventional biofilm formation—Flo8, Gal4 and Rfx2—have not yet been explored in terms of sexual biofilm formation and is another area of interest for future research. Finally, the transcription factor Cph1, which is not required for conventional biofilm formation, plays a central role in the regulation of sexual biofilm formation [58,60]. Deletion of *CPH1* results in the complete obliteration of sexual biofilm formation, and it has been hypothesized that Cph1 is the terminal transcription factor activated by the MAPK cascade in both white and opaque cells responding to pheromone [58]. These ideas have been challenged, where another group found that although the same GPCR (Ste2/3), MAPK cascade (Ste11, Hst7, Cek1/2) and scaffolding protein (Cst5) are used in both white and opaque cell pheromone responses, there are cell type differences in the terminal transcription factors that are activated by pheromone [74]. In opaque cells, their findings suggest that Cph1 is activated for mating, while in white cells, Tec1 is activated for sexual biofilm formation [74,119,120]. The discrepancies between these two findings may be partially explained by differences in growth conditions utilized by the two groups [11,12,58,74,86]. In fact, the different conditions lead to the formation of sexual biofilms with distinct structural features, and one possibility is that different transcription networks may be involved in the two conditions that depend on distinct environmental cues. Given this information, the terminal transcription factor(s) activated by pheromone-stimulated MAPK signaling in white cells remain to be conclusively determined.

The transcriptional network regulating conventional biofilms has been shown to have evolved fairly recently [60]. By determining the master regulators of sexual biofilm formation and its accompanying transcriptional network, we will be able to explore how two seemingly unrelated transcriptional networks and signaling pathways have evolved to interact with one another. If Cph1 is the terminal transcription factor of the pheromone response in white cells, this would indicate that a conserved signaling cascade and its transcriptional regulator evolved to control a novel set of genes during pheromone activation. We can envision two scenarios where this could occur. First, genes associated with biofilm formation may have come under the direct control of Cph1 by the addition of Cph1 recognition sequences to their promoters. Alternatively, one or several regulators of biofilm formation may have come under the control of Cph1 [115]. In the latter scenario, deemed the “regulator-first” model of the evolution of transcription networks [115], Cph1 would have been directly inserted into the older conventional biofilm network, gaining control of several downstream genes associated with biofilm formation, while adding many of the genes that it previously regulated to the network. This model could account for the large size of transcriptional networks (e.g., the conventional biofilm network comprises approximately 20% of the genome), and the reason why complex transcriptional networks include such large numbers of seemingly extraneous target genes [58,60,115]. Since white cells are unable to mate, their main purpose is to form biofilms in response to pheromone, thus reason dictates that they have no need to express genes involved in mating when stimulated by pheromone. Yet, the expression of mating genes has been observed in white cells responding to mating pheromone, where there is a clear induction of genes involved in cell fusion, karyogamy and other aspects of mating (e.g., *FUS1* and *KAR4*) [58]. This regulator-first model is consistent with the hypothesis that Cph1 is the terminal transcription factor activated by the MAPK cascade in both white and opaque cells responding to pheromone. In the alternative hypothesis, Tec1, whose expression is only induced in conventional biofilms via Efg1, may have come under direct control of a novel signaling pathway, namely the pheromone response MAPK cascade. In this scenario, Tec1 would still regulate many of the genes it traditionally regulated and the transcriptional profile of the white cell pheromone response would look similar to conventional biofilm formation. Given that we see a dramatic change in transcriptional profiles between the two biofilm systems and the activation of so many extraneous genes involved in mating in white cells responding to pheromone, we favor the regulator-first model for the evolution of the sexual biofilm transcriptional network.

## 5. Conclusions

Sexual biofilms represent a specialized kind of biofilm formed by *MTL-*homozygous cells responding to mating pheromone. The physical characteristics of sexual biofilms differ dramatically from conventional biofilms; indeed, they appear to lack the major characteristics that contribute to the highly pathogenic nature of conventional biofilms. The molecular differences that result in such distinct phenotypes between the two systems remain to be determined. The significance of the unusual characteristics of sexual biofilms and their roles in the lifecycle of *C. albicans* is also not clearly understood. The low frequency of *MTL-*homozygous strains observed in nature and the apparent lack of opaque-specific niches outside of the laboratory led to questions about the existence of a parasexual lifecycle in *C. albicans* in nature. However, it is now appreciated that sexual biofilms may serve as a permeable and penetrable, yet protective, microenvironment that promotes mating in *C. albicans*. Although no in vivo model has been established to investigate the relevance of sexual biofilms in the host, the apparent disadvantageous properties of sexual biofilms for survival in the host may be outweighed by their ability to promote parasexual mating. Future work on the genetic regulation and molecular mechanisms of sexual biofilm formation will improve our understanding of the significance of sexual biofilms as well as the relevance of phenotypic switching and parasexual mating in the lifecycle of *C. albicans* in nature. Overall, the molecular and genetic regulation of conventional and sexual biofilm formation is quite different between the two systems. Conventional biofilms are modulated by the Ras1/cAMP signaling pathway, whereas sexual biofilms are modulated by a MAP kinase pathway; each activating a largely distinct set of transcription factors and likely different transcriptional networks. Understanding how these two transcriptional networks regulate their target genes to give rise to similar yet distinct phenotypes will also provide a basis for studies on the evolution of biofilm formation. Current and future research into sexual biofilms should provide a wealth of knowledge into the molecular genetics, pathogenesis, and evolutionary history of one of the most pervasive fungal pathogens of humans.
